# Secondary and Co-Infections in Hospitalized COVID-19 Patients: A Multicenter Cross-Sectional Study in Malaysia

**DOI:** 10.3390/antibiotics12101547

**Published:** 2023-10-16

**Authors:** Siti Roszilawati Ramli, Fashihah Sherina Abdul Hadi, Nur Asyura Nor Amdan, Insyirah Husna Kamaradin, Noraliza Zabari, Saraswathiy Maniam, Nur Suffia Sulaiman, Sumarni Ghazali, Zamtira Seman, Rohaidah Hashim, Norazah Ahmad

**Affiliations:** 1 Infectious Diseases Research Centre, Institute for Medical Research, National Institutes of Health, Setia Alam, Shah Alam 40170, Malaysia; 2 Nutrition, Metabolism & Cardiovascular Research Centre, Institute for Medical Research, National Institutes of Health, Setia Alam, Shah Alam 40170, Malaysia; 3 Special Resource Centre, Institute for Medical Research, National Institutes of Health, Setia Alam, Shah Alam 40170, Malaysia; 4 Sector for Biostatistics & Data Repository, National Institutes of Health, Setia Alam, Shah Alam 40170, Malaysia

**Keywords:** COVID-19, antibiotic usage secondary infection, co-infection, bacteria, fungal, Malaysia

## Abstract

Bacterial and fungal secondary and co-infections are commonly identified with viral respiratory infections. This study was undertaken to determine the incidence and factors associated with bacterial and fungal infections in patients with COVID-19 as well as antibiotics prescription patterns within the first and second waves of the outbreak in Malaysia. Clinical records of 3532 COVID-19 patients admitted to hospitals in Malaysia between 4 February and 4 August 2020 were analyzed. Co-morbidities, clinical features, investigations, treatment, and complications were captured using the REDCap database. Culture and sensitivity test results were retrieved from the WHONET database. Univariate and multivariate regression analyses were used to identify associated determinants. A total of 161 types of bacterial and fungal infections were found in 81 patients, i.e., 2.3%. The most common bacterial cultures were Gram-negative, i.e., *Pseudomonas aeruginosa* (15.3%) and *Klebsiella pneumoniae* (13.9%). The most common fungal isolate was *Candida albicans* (41.2%). Augmentin, ceftriaxone, tazocin, meropenem, and azithromycin were the five most frequently prescribed antibiotics. The latter four were classified under the “Watch” category in the WHO AwaRe list. Our data showed that bacterial and fungal secondary and co-infections were frequently found in severely ill COVID-19 patients and were associated with a higher mortality rate.

## 1. Introduction

A serious acute respiratory infection outbreak emerged in Wuhan, China, in December 2019, which led to a global pandemic. The disease was named nCOV-19, which was later changed to coronavirus disease 2019 or COVID-19 [1]. The novel beta coronavirus, severe acute respiratory syndrome coronavirus 2 (SARS-CoV-2), was first isolated from a bronchoalveolar lavage sample of an infected patient and was identified as the causative agent of this infection [2].

COVID-19 is an infection that can cause both pulmonary and systemic inflammation, leading to multi-organ dysfunction in patients at high risk [3]. Those at high risk of developing severe symptoms if infected with this virus include persons with heart or lung disease, people with poor immune systems, and the elderly [4]. Acute respiratory distress syndrome and respiratory failure, sepsis, acute cardiac injury, and heart failure were the most common critical complications during exacerbations of COVID-19 [5,6].

COVID-19 patients may also exhibit co-infections or develop secondary infections, especially critically ill patients who are more susceptible to those infections [7]. Secondary bacterial infections were also observed in 4 out of 13 patients in intensive care units (ICU) (31%) and in 10% of patients overall [8,9]. In a study in Italy, 8.5% of 3200 COVID-19-related deaths from various regions that were the subject of an Italian investigation had secondary illnesses [10]. It was reported that 10% to 30% of patients with severe COVID-19 and managed in ICU were infected with secondary bacterial and fungal infections. The observed occurrence can be ascribed to the cumulative influence of immunosuppressive agents in conjunction with viral infection. Predominant origins of secondary or co-infections encompass pneumonia, bacteremia, and catheter-related infections [7].

Historically, the majority of deaths associated with the Spanish Flu pandemic in 1918 were speculated to be related to bacterial pneumoniae rather than the infection caused by the virus itself [11]. Secondary infections caused by bacteria were also associated with high morbidity and mortality in the influenza pandemic in 1957, 1968, and 2009. During the 2009 swine flu pandemic, 29% to 55% of deaths were caused by secondary bacterial infections [12,13]. In a study of 150 cases of COVID-19 in Wuhan, as many as 11 (16%) of the 68 cases of reported mortality had secondary infections [14].

The first wave of the COVID-19 epidemic in Malaysia began in late January 2020, while the second wave started in late February 2020 and lasted for approximately three weeks [15]. The first three cases of COVID-19 in Malaysia were imported and reported on 25 January 2020. The cases were detected during the screening of close contacts of a confirmed case of a Chinese nationality in Singapore who had travelled into Malaysia via Johor. The first case of COVID-19 in a Malaysian citizen was confirmed in early February 2020 as the ninth case overall [16].

This first wave lasted for almost three weeks, and the total number of cases was considered low with full recovery. Twenty-two cases were reported, with twenty imported cases and two local transmission cases, and no deaths [17,18]. After an eleven-day hiatus, a second wave commenced on 27 February 2020 due to a religious gathering in Kuala Lumpur, Malaysia, which was held from 27 February to 1 March 2020 and attended by 14,500 attendees. The presented cases were classified into several clusters, and the biggest one was the Sri Petaling tabligh cluster, with a 6.5% infection rate that contributed to 47% of all cases in Malaysia [17]. On 15 March 2020, the number of daily new cases escalated from 41 to 190 cases, and Malaysia recorded the first three-digit daily new cases after two weeks of the gathering. As the number of new cases kept increasing and climbed to 553 cases, the first national partial lockdown or Movement Control Order (MCO) was implemented by Malaysian authorities on 18 March 2020 to reduce social mixing and to contain the virus. This was then followed by a stricter version called enhanced MCO on 27th March. On 4th May, the enhanced MCO was replaced by the Conditional Movement Control Order (CMCO) following a decline in cases. The less strict MCO was then announced as Recovery Movement Control Order (RMCO) on 10 June 2020 [15].

While there are some published data on bacterial infections, there appears to be a lack of publications on fungal infections among COVID-19 patients [19,20,21,22]. Due to the paucity of data and literature pertaining to bacterial and fungal secondary and/or co-infections in COVID-19 patients within the context of Malaysia, the primary objective of this investigation is to assess and establish the prevalence of bacterial and fungal secondary and/or co-infections in the cohort of hospitalized individuals diagnosed with COVID-19. Furthermore, our research aims to conduct an assessment of the risk factors associated with bacterial and fungal infections in addition to identifying the predominant pathogens responsible for secondary and co-infections in COVID-19 patients. Additionally, we seek to delineate the corresponding treatment approaches employed for managing these infections. This comprehensive analysis aims to contribute valuable insights into the understanding of fungal and bacterial infections in COVID-19 patients, thereby guiding healthcare practitioners in making critical and informed decisions concerning the management of such cases.

## 2. Results

A total of 3532 COVID-19 patients’ data were analyzed. A total of 161 types of bacterial and fungal infections were found in 81 patients, which is an overall prevalence of 2.3%. There was an increasing trend of co-infections and/or secondary infections from the youngest to the oldest group. Those 30 years and under had a co-infection and/or secondary infections rate of 0.7%, while those above 70 years had a rate of 7.4%. There were similar rates among both genders, 2.3%. The incidence rates between races were not significantly different. Table 1 summed up demographic, clinical characteristics, and laboratory investigations of COVID-19 patients with secondary and/or co-infections with bacterial and/or fungal infections.

A summary of univariate and multivariate analyses for clinical characteristics associated with increased risks for bacterial and fungal secondary and/or co-infections in COVID-19 patients is displayed in Table 2. In the univariate analyses, those with secondary and/or co-infections were more likely to be older and symptomatic, which was reported as fever, cough, shortness of breath, vomiting/nausea, and diarrhea. Abnormal chest X-ray findings and high respiratory rates were also associated with secondary and co-infections. They were also more likely to have severe symptoms (COVID-19 stages 4 and 5). Those with secondary and/or co-infections significantly had a higher risk for mortality and longer hospital admissions. Co-morbidities associated with higher secondary and co-infections in patients included chronic kidney disease, hypertension, obesity, and diabetes mellitus. 

In the multivariate analyses, COVID-19 patients with bacterial and fungal secondary and co-infections were significantly associated with increasing age, raised systolic blood pressure (>160 mmHg), and co-morbidities such as diabetes, hypertension, chronic hematological disease, and chronic kidney disease. Those with secondary and/or co-infections were more likely to suffer from the complications of acute respiratory distress syndrome (AOR = 7.7, 95% CI: 3.9, 15.1) and acute renal failure (AOR = 9.6, 95% CI: 5.2, 18.0). Those with fungal/bacterial infections were twice as likely to report having a fever (AOR = 2.2, 95% CI: 1.3, 3.8). 

Laboratory parameters among patients with secondary and/or co-infection compared to those without, showed that all parameters were significantly different between the two groups except for white blood cell count (Table 3). Laboratory investigations significantly associated with secondary and/or co-infections in patients were hemoglobin level, lymphocyte count, neutrophil count, platelet count, D-dimer, C-reactive protein (CRP), procalcitonin (PCT), ferritin, and lactate dehydrogenase (LDH).

**Figure 1:**
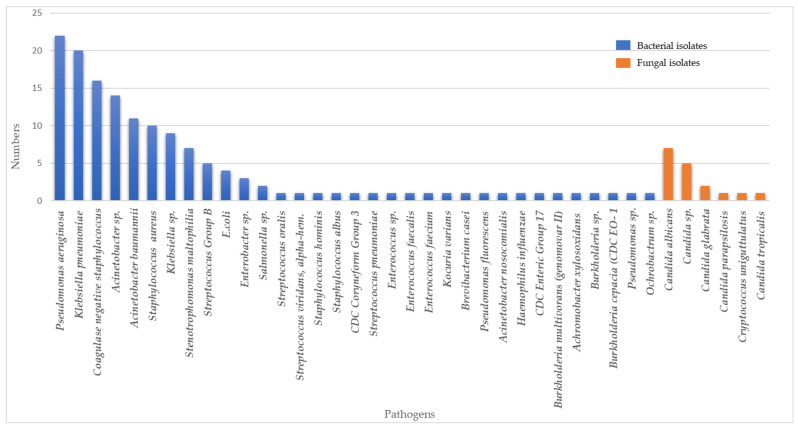
Composition and distributions of bacterial and fungal pathogens in COVID-19 patients with secondary and/or co-infections.

A total of 39 types of pathogens were isolated from COVID-19 patients within the study period (Figure 1). Most isolates were Gram-negative bacteria (102, 63%), followed by Gram-positive bacteria (42, 26%) and fungi (17, 11%). The most common bacterial pathogens were *Pseudomonas aeruginosa (P. aeruginosa)* (22/161, 13.6%), *Klebsiella pneumoniae (K. pneumoniae)* (20/161,12.4%), coagulase-negative *Staphylococcus* (16/161, 9.9%), *Acinetobacter* spp. (14/161, 8.6%), and *Acinetobacter baumannii (A. baumanii)* (11/161, 6.8%). *Candida albicans* (7/161, 4.3%), *Candida* spp. (5/161, 3.1%), and *Candida glabrata* (2/161, 1.2%) were the three most frequent fungi isolated. Six out of twenty *K. pneumoniae* and one out of nine *Klebsiella* spp. were classified as extended-spectrum beta-lactamase (ESBL), two out of twenty *K. pneumoniae* were carbapenem-resistant Enterobacterales (CRE), six out of eleven *A. baumanii*, and five out of fourteen *Acinetobacter* spp. were multidrug-resistant (MDR), and one out of ten *Staphyloccocus aureus* (*S. aureus*) were methicillin-resistant *S. aureus* (MRSA).

**Figure 2:**
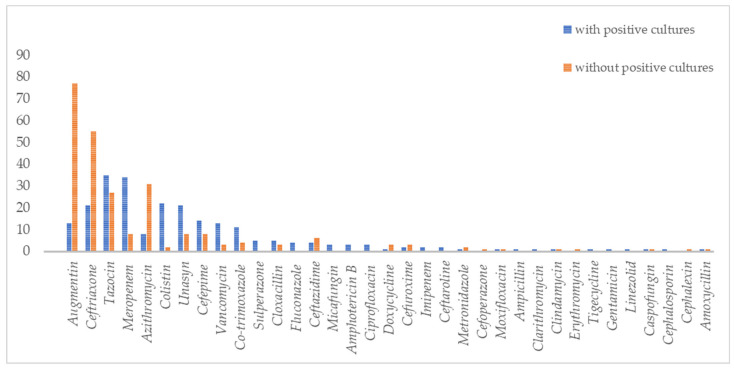
Types of antibiotics and antifungals used in COVID-19 patients with and without bacterial and fungal co-infections.

In terms of prescribing antibiotics patterns amongst COVID-19 hospitalized patients, 6.9% (*n* = 245) received antibiotics/antifungal treatment. Out of those treated, 73% did not have an obvious source of bacterial or fungal infections. Augmentin, ceftriaxone, tazocin, meropenem, and azithromycin were the five most frequently prescribed antibiotics for COVID-19 patients (Figure 2). The five most common antibiotics used among patients with positive secondary/co-infection bacterial cultures were tazocin (35/237, 15%), meropenem (34/237, 14%), colistin (22/237, 9%), unasyn (21/237, 9%), and ceftriaxone (21/237, 9%). At the same time, the five most common antibiotics used as empirical antibiotic therapy were augmentin (77/247, 31%), ceftriaxone (55/247, 22%), tazocin (27/247, 10%), azithromycin (12/247, 12%), and cefepime (8/247, 3%). Glucocorticoids were used in 36.9% (1045/2834) of patients.

**Figure 3:**
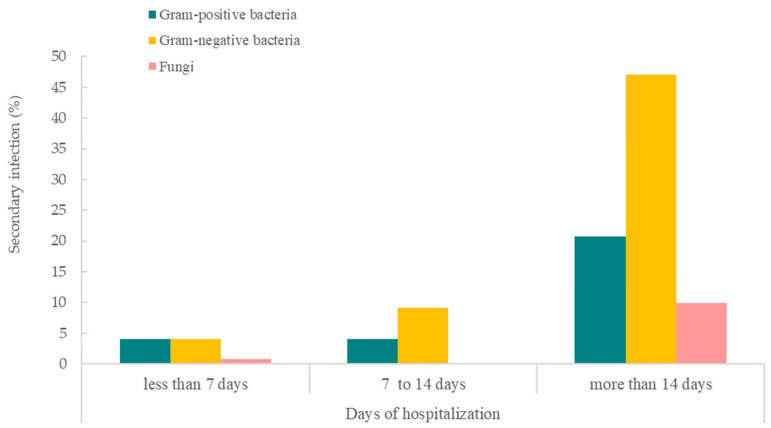
Days of hospitalization of COVID-19 patients with bacterial and fungal secondary and/or co-infections.

Figure 3 shows that COVID-19 patients with hospitalization of more than 14 days had a higher percentage of bacterial or fungal secondary and/or co-infections. Among the pathogens, Gram-negative bacteria were the main type of infection among patients hospitalized for more than 14 days (*n* = 57; 47.1%) and also for 7 to 14 days (*n* = 11; 9.1%). While there was a low secondary/co-infection rate in patients hospitalized for less than 7 days, similar percentages of Gram-positive (*n* = 5; 4.1%) and negative bacterial infections (*n* = 5; 4.1%) were observed. Gram-positive bacteria were observed to be the second most common cause of infections for more than 14 days of hospitalization (*n* = 25; 20.7%) and within 7 to 14 days of hospitalization (*n* = 5; 4.1%). Fungi, however, was the least common cause of secondary/co-infection for COVID-19 patients hospitalized for more than 14 days (*n* = 12; 9.9%) and less than 7 days (*n* = 5; 4.1%). No secondary/co-infection with fungi was reported in the 7 to 14 days hospitalization group.

**Figure 4:**
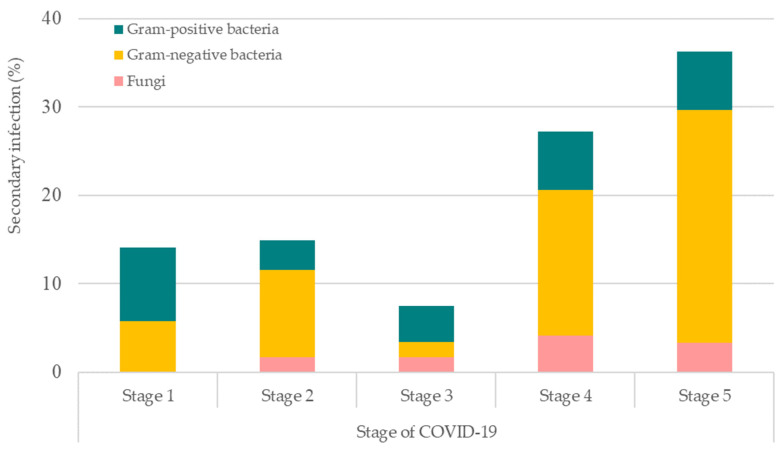
Stages of COVID-19 patients that were inflicted with bacterial and fungal secondary and/or co-infections. Stage 1: asymptomatic; Stage 2: symptomatic with pneumoniae; Stage 3: pneumoniae without hypoxia; Stage 4: pneumoniae with hypoxia requiring oxygen supplementation therapy; and Stage 5: critically ill.

We further evaluated the stages of COVID-19 patients that were inflicted with bacterial and/or co-infections (Figure 4). Stage 5 had the highest rate of COVID-19 patients inflicted with such infections, followed by stage 4, stage 2, stage 1, and stage 3. Among these, Gram-negative bacteria were the most common cause of infections for secondary and/or co-infections in stage 5, 4, and 2 COVID-19 patients, followed by Gram-positive bacteria and fungi. However, Gram-positive bacteria were observed to be the most prominent cause of infection for stages 1 and 3 of COVID-19 patients.

**Figure 5:**
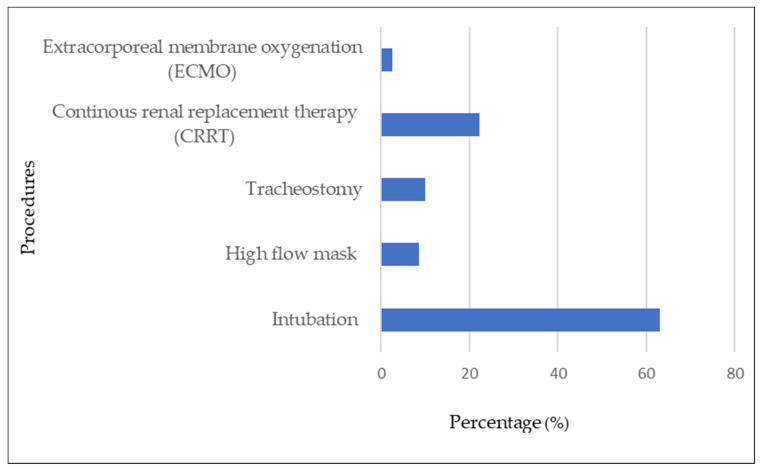
Procedures associated with COVID-19 patients with bacterial and fungal secondary and/or co-infections.

Most of the COVID-19 patients who had bacterial/fungal infections received various supportive therapies. Tracheal intubation was the most commonly performed procedure, that is, in 63% (51/81) of these patients. Other procedures were continuous renal replacement therapy (CRRT) (18/81; 22.2%), tracheostomy (8/81; 9.9%), high-flow mask (7/81; 8.6%), and extracorporeal membrane oxygenation (ECMO) (2/81; 12.5%) (see Figure 5). All procedures were significantly associated with secondary and/or co-infections. Bacterial/fungal infections were present in those who had tracheal intubation (85%), tracheostomy (47%), CRRT (46.2%), ECMO (25%), and high-flow mask (6.4%).

**Figure 6:**
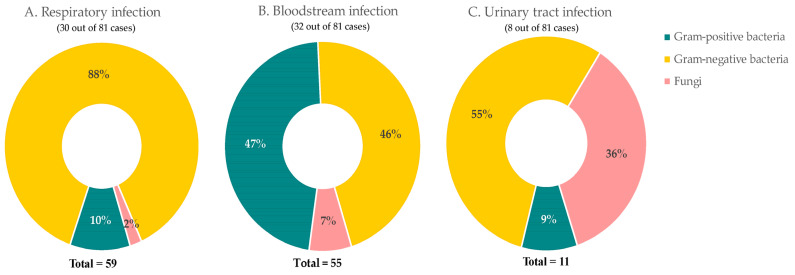
Common sites of bacterial and fungal co-infections in COVID-19 patients. Percentage of composition of pathogens in (**A**) respiratory, (**B**) bloodstream, and (**C**) urinary tract.

Overall, 81 (2.3%) patients contracted co-infections. The main infection sites were blood (32 cases), respiratory (30 cases), and urinary tract (8 cases). A total of 59 pathogens were identified (Figure 6A) in respiratory infection patients, which were mostly Gram-negative bacteria (52 cases, 88%) followed by Gram-positive bacteria (6 cases,10%) and fungi (1 case, 1.7%). A total of 55 pathogens were detected in 32 patients with bloodstream infections (Figure 6B). Twenty-six (47%) infections were caused by Gram-positive bacteria, 25 (46%) by Gram-negative bacteria, and four fungi (7%). While for urinary infections (Figure 6C), 11 pathogens were isolated from eight patients. They were caused by six (55%) Gram-negative bacteria, four (36%) fungi, and one (9%) Gram-positive bacteria.

## 3. Materials and Methods 

### 3.1. Study Design

This is a multicenter observational retrospective study supported by the National Institutes of Health, Malaysia, in collaboration with infectious diseases teams and clinical research centers. We included 3532 patients from ten designated COVID-19 hospitals from 22 January to 30 April 2020. Hospitals included in this study were Hospital Kuala Lumpur, Hospital Melaka, Hospital Pulau Pinang, Hospital Raja Perempuan Bainun, Hospital Raja Perempuan Zainab II, Hospital Sultanah Bahiyah, Hospital Sultanah Nur Zahirah, Hospital Sungai Buloh, Hospital Tengku Ampuan Afzan, and Hospital Tuanku Jaafar.

### 3.2. Data Source and Data Selection

All reverse transcriptase–polymerase chain reaction (RT-PCR)-confirmed COVID-19 patients aged one year and up were recruited sequentially depending on complete results at discharge. A nationwide ClinData COVID-19 registry was established utilizing REDCap technology, which has been revised from the International Severe Acute Respiratory and Emerging Diseases Registry.

Infection Consortium (ISARIC)–WHO Case Report Form was used for data entry [23,24]. Data verification was performed with standardized protocol prior to submission into the ClinData COVID-19 registry. Culture and antibiotic sensitivity test results were retrieved from Malaysian WHONET database. WHONET is a national database that collects, collates, and analyzes the antimicrobial susceptibility data of all clinical isolates in Malaysia [25].

The inclusion criteria for the study are patients who were 18 years and above and admitted to hospital for COVID-19 between 22 January and 30 April 2020 with confirmed culture and sensitivity results (recorded in WHONET database) within the date of admission and discharge of the respective patient. The exclusion criteria were COVID-19 patients with positive culture and sensitivity results outside the range of the dates of admission and discharge.

Out of 3532 COVID-19 patients captured in the REDCap database, 81 (2.23%) patients were found to have positive culture and sensitivity tests in the WHONET database. The search was based on the presence of similar identification card number/passport number and name between both databases within the dates of admission and discharge of the respective patients. The data were merged and checked by three independent individuals.

### 3.3. Laboratory Confirmation

COVID-19 cases were diagnosed in accordance with the interim guidelines provided by the World Health Organization (WHO). All individuals included in the study exhibited positive results in their reverse-transcriptase–polymerase chain reaction (RT-PCR) tests, which targeted the detection of SARS-CoV-2. Various types of samples, such as nasopharyngeal and/or oropharyngeal swabs, tracheal aspirates, sputum, or serum, were collected from the patients and subjected to RT-PCR analyses. These analyses were conducted at designated National Public Health Laboratories, Institute for Medical Research, and accredited hospital laboratories specializing in COVID-19 diagnoses. Additionally, culture samples of lower respiratory specimens, urine, catheters, and blood were collected from the patients for etiology examinations. Endotracheal aspirate samples were obtained specifically from patients receiving invasive mechanical support, such as tracheal intubation or tracheotomy. Moreover, all individuals suspected of having sepsis and possessing indwelling catheters underwent simultaneous examination of peripheral venous and catheter blood cultures. The presence of a bacterial or fungal infection was determined by positive laboratory-confirmed etiological results (culture positive) following admission. Bacterial and fungal infections were classified as either co-infections (acquired within the community or within 3 days of hospital admission) or secondary infections (developed after a hospital stay exceeding 3 days) [26].

### 3.4. Disease Staging and Clinical Management

Patients who were admitted to designated hospitals were under the care of infectious diseases teams, following the COVID-19 Management Guideline established by the Ministry of Health in Malaysia (MOH, 2020). The patients were categorized into different stages based on their clinical severity: stage I denoted asymptomatic cases; stage II represented symptomatic cases without pneumonia; stage III indicated pneumonia without hypoxia; stage IV encompassed pneumonia with hypoxia necessitating oxygen supplementation therapy; and stage V encompassed critically ill patients. Vigilant monitoring for early signs of deterioration and the implementation of appropriate and proactive interventions were part of the standard protocol. As outlined in the guideline, all patients were hospitalized for a duration of 14 days or upon testing negative for SARS-CoV-2 in repeated nasopharyngeal/oropharyngeal swabs or in the event of mortality.

### 3.5. Study Variables and Outcome of Interest

Variables related to demographic data, clinical features, co-morbidities, laboratory and radiographic investigations, antibiotic treatments, and clinical outcomes were analyzed for their associations with bacterial and fungal secondary and/or co-infections.

### 3.6. Statistical Analyses

Data from patients were identified and evaluated as a group. Continuous measurements were provided as the median and interquartile range, whilst categorical variables were described as frequency and percentage. The Mann–Whitney U test and Fisher’s exact test were used to assess differences in COVID-19 patient severity. To investigate risk factors related to disease severity, univariate, and multivariate logistic regressions were performed. For all analyses in this investigation, the two-sided statistical significance level, *p*-value, was fixed at 0.05 using SPSS version 3.6.3 software.

### 3.7. Study Ethics

The study was registered with the National Medical Research Register (NMRR-20-2002-56396 (IIR)) and was authorized by the Malaysian Ministry of Health’s Medical Research and Ethics Committee (KKM/NIHSEC/P20-2287 (4)).

## 4. Discussion

This study found a total of 81 cases, i.e., a 2.3% incidence rate of secondary and/or co-infections among COVID-19 patients. There were also low incidence rates in other studies. A study in the United States reported a 4.8% bacterial co-infection rate, while studies in China reported rates from 1–4% [19,27]. In a meta-analysis of hospitalized ICU and non-ICU COVID-19 patients, 7% had bacterial co-infection [1]. The incidence of bacterial and fungal infections in our study was likely to be under-diagnosed as they were based on positive microbiologic test results. There may have been a lesser chance of obtaining positive microbial cultures after antibiotics have been started in patients or due to inadequate blood volume or low quality of sputum specimens for microbial cultures [28,29,30].

Our findings showed that Gram-negative bacteria accounted for the secondary/co-infections in COVID-19 patients. These findings correlated with Wang et al. (2020) and Chen et al. (2020), which reported both multiple Gram-negative bacteria and fungal organisms in patients infected with COVID-19 [14,27,28,29,30]. A study by Vaillancourt et al. 2020 observed that *P. aeruginosa* was detected only in symptomatic patients regardless of disease severity [31]. Grasseli et al. 2020 reported that *P. aeruginosa*, *Enterobacter* spp., and *Escherichia coli* were the most prevalent bacteria involved in respiratory tract infections in COVID-19 patients [32]. However, Nebreda-Mayoral et al. 2022 found that multidrug-resistant *A. baumannii* was observed as the most common agent of respiratory superinfection in 16% of COVID-19 patients [33]. In comparison, Said et al. (2022) reported that *K. pneumoniae* was the most prevalent bacteria isolated in both respiratory and urinary tract infections among COVID-19 patients [34].

*P. aeruginosa* and *K. pneumoniae* were common causes of co-infection and secondary infection in COVID-19 cases in the United Kingdom [35]. In this study, *P. aeruginosa* was noted to be the most common bacteria isolated in blood, while *K. pneumoniae* was commonly associated with severe respiratory tract infections. Our study showed that *P. aeruginosa* and *K. pneumoniae* were the two main bacteria associated with high mortality in secondary and/or co-infections, i.e., 35% (6/17) and 27% (4/15) mortality, respectively. *Candida* spp. was commonly associated with infections in the female genital tract [36]. However, in this study, *Candida* spp. was found in urine and catheter samples from both men and women. Among those with *Candida* spp. infections, 30% (3/10) of the patients did not survive. These findings aligned with an earlier report which indicated that infections caused by *Candida* non-*albicans* had a high mortality risk [37].

In this study, some confirmed COVID-19 cases were hospitalized for 14 days for treatment and observation. This increased the chances of acquiring hospital-acquired infections among patients with high risks. Factors that were significantly associated with bacterial and fungal co-infections and/or secondary infections included fever, stage 5 COVID-19, chronic kidney disease, hypertension, systolic blood pressure >140 mmHg, chronic hematology disease, diabetes mellitus, acute respiratory distress syndrome, acute renal failure, and increased age. Procedures such as tracheal intubation, tracheostomy, high-flow mask, CRRT, and ECMO were also significantly associated with such co-infections. A study by Zhang et al. (2020) observed secondary infection risks increased after receiving invasive respiratory ventilation and intravascular devices, which led to poorer outcomes. They reported respiratory infection rates of 92.31% in tracheotomy, 30.43% in tracheal intubation, and 12.90% in high-flow procedures [38]. Zhou et al. (2020) reported that ventilator-associated pneumonia occurred in 31% of patients. Those with secondary/co-infections also had a significantly higher risk for mortality and longer hospital admissions. [12,39]

For laboratory findings, nine out of ten parameters which included inflammatory markers, were significantly different between patients with bacterial and fungal secondary/co-infections and those without. This was also seen in a meta-analysis which showed elevated serum CRP, PCT, D-dimer, and ferritin among COVID-19 patients were associated with a poor outcome [9]. However, elevated inflammatory serological markers that are often linked to bacterial infection, such as elevated PCT and CRP, are not specific and can also occur without such infection [1].

This study denoted that antibiotics were prescribedbased on recommended guidelines, i.e., augmentin, ceftriaxone, and azithromycin were highly used empirically. Augmentin and azithromycin were recommended to be used as an empirical treatment in mild and atypical pneumonia, respectively [40]. Macrolides, cephalosporins (especially third- and fourth-generation cephalosporins), and fluoroquinolones were other popular classes of antibiotics for treating COVID-19 patients. The five most frequently prescribed antibiotics are all on the “Watch” category of the WHO AWaRe classifications list [41]. The only exception was augmentin which falls under the “Access” category. The next five most-prescribed antibiotics were unasyn, co-trimoxazole, cefepime and vancomycin (from the “Watch category), and colistin (“Reserve” category). It was noted that colistin was strictly prescribed for the few multidrug-resistant *A. baumanii* cases and reported as a last-resort treatment. The frequent use of tazobactam/piperacillin was justified by the many cases of *P. aeruginosa* infections. The use of broad-spectrum antibiotics should be de-escalated once cultures identify specific organisms [42,43].

As this study was conducted during the first and second waves of the COVID-19 outbreak in Malaysia, there were no established protocols for treating bacterial or fungal infections associated with COVID-19 disease at that time. The use of antibiotics was based on current practice and limited understanding of COVID-19. Nevertheless, judicial use of antibiotics by balancing the need for antibiotic administration with knowledge of the risk of secondary and/or co-infection in hospitalized COVID-19 patients remain essential and crucial. Furthermore, the incidence of multidrug-resistant bacterial and fungal infections among COVID-19 patients should be monitored closely as the rise may cause high-risk patients to become more susceptible and more difficult to manage during viral pandemics [44]. Secondary and co-infections in COVID-19 patients with other respiratory pathogens can contribute to more serious complications and worsen clinical severity. Early detection of these infections may be beneficial for patients’ prognosis and management, preventing unnecessary use of antimicrobial agents.

To our knowledge, this is the first report on bacterial/fungal infections in COVID-19 patients managed in tertiary hospitals in Malaysia. The strengths of the study included the use of a comprehensive dataset from ten designated COVID-19 hospitals. The limitations of this study, however, should be acknowledged. The data on bacterial and fungal infections were incorporated from a national database, which reduced the number of samples based on the availability of complete data from both databases. Although the former database has a high degree of completion, it is possible that some testing and reporting were reduced during the COVID-19 response, which partially explained the lack of laboratory data that contributed to unrecorded isolate results in COVID-19 cases. The small size of the population with secondary and co-infections and incomplete patient clinical details caused limitations in fully differentiating between these two types of infections. There could also be missing data due to limited testing for secondary and co-infections among asymptomatic and less severe COVID-19 cases. 

Future studies could use a more robust study design to conduct screening for bacterial/fungal co-infection or secondary infections in a cohort of COVID-19 patients. Opportunities for improvement include indicating the temporal relationship of bacterial and fungal infection relative to patient presentations and antibiotics administration. Ideally, prospective cohort studies could include blood and other specimen collections from COVID-19 patients, including those attended at healthcare centers as well as community clinics. 

## 5. Conclusions

The overall incidence of bacterial/fungal secondary and/or co-infection rate among COVID-19 patients in Malaysia in the early phase of the outbreak was relatively low compared to that reported by other countries. Elderly persons and those with co-morbidities such as diabetes, hypertension, chronic kidney disease, acute respiratory distress syndrome, and acute renal failure were more susceptible to bacterial/fungal secondary and co-infections. Timely and accurate evaluations for bacterial or fungal secondary and co-infections in viral infections are crucial, enabling the prompt initiation of appropriate antibiotic therapies when necessary. Embracing such a proactive strategy empowers healthcare providers to play a substantial role in enhancing patient outcomes and achieving more efficient disease management.

## Figures and Tables

**Table 1:** Demographic and clinical characteristics and laboratory investigations of COVID-19 patients with secondary and/or co-infections with bacterial and/or fungal infections.

Variables		With Bacterial/ Fungal Infection	Without Bacterial/ Fungal Infection	*p*-Value
		*n* = 81 (2.23%)	*n* = 3451 (97.7%)	
**Demography**				
Age group (years)				<0.001 ^a^
	<31	12 (0.7)	1618 (99.3)	
	31–50	13 (1.2)	1030 (98.8)	
	51–70	49 (6.4)	715 (93.6)	
	71+	7 (7.4)	88 (92.6)	
Gender				0.947 ^a^
	Female	21 (2.3)	906 (97.7)	
	Male	60 (2.3)	2545 (97.7)	
Race				<0.001 ^b^
	Malay	66 (3.1)	2072 (96.9)	
	Chinese	3 (1.5)	191 (98.5)	
	Indian	4 (3.4)	113 (96.6)	
	Others (Sabahan, Sarawakian, Orang Asli, foreigners)	8 (0.7)	1075 (99.3)	
**Exposure and medical history**				
History of travel				0.064 ^a^
	Yes	19 (1.6)	1148 (98.4)	
	No	62 (2.6)	2303 (97.4)	
Co-morbidities				<0.001 ^a^
	Bronchial asthma	5 (4.5)	107 (95.5)	
	Hypertension	46 (8.4)	502 (91.6)	
	Chronic kidney disease	17 (23.6)	55 (76.4)	
	Malignancy	1 (6.3)	15 (93.8)	
	Chronic hematological disorder	1 (14.3)	6 (85.7)	
	Obesity	3 (11.1)	24 (88.9)	
	Diabetes mellitus	39 (10.7)	326 (89.3)	
	Smoking	7 (1.9)	364 (98.1)	
	History of taking medications	39 (6.3)	585 (93.8)	
**Clinical features**				
Symptoms				<0.001 ^a^
	Fever	52 (5.3)	929 (94.7)	
	Cough	48 (4.4)	1037 (95.6)	
	Runny nose	9 (2.5)	348 (97.5)	
	Shortness of breath	22 (11.6)	168 (88.4)	
	Nausea/vomiting	5 (8.2)	56 (91.8)	
	Diarrhea	15 (7.9)	176 (92.1)	
	Skin rashes	1 (16.7)	5 (83.3)	
Respiratory rate (per minute)				<0.001 ^b^
	<20	52 (1.6)	3262 (98.4)	
	>20	29 (13.3)	189 (86.7)	
Temperature on admission (°C)				<0.001 ^a^
	<37.5	57 (1.7)	3218 (98.3)	
	>37.5	24 (9.3)	233 (90.7)	
Chest X-ray findings				0.001 ^a^
	Abnormal findings	38 (3.6)	1017 (96.4)	
	Normal findings	43 (1.7)	2434 (98.3)	
Systolic blood pressure (mmHg)				0.008 ^a^
	<140	50 (2.2)	2174 (97.8)	
	>140	31 (4.0)	737 (96.0)	
Diastolic blood pressure (mmHg)				0.964 ^a^
	<90	72 (2.3)	3062 (97.7)	
	>90	9 (2.3)	389 (97.7)	
Complications				<0.001 ^b^
	Seizure	1 (20.0)	4 (80.0)	
	Stroke	3 (50.0)	3 (50.0)	
	Congestive heart failure	4 (22.2)	14 (77.8)	
	Cardiac arrhythmia	11 (37.9)	18 (62.1)	
	Cardiac arrest	7 (28.0)	18 (72.0)	
	Disseminated intravascular coagulopathy	5 (41.7)	7 (58.3)	
	Acute kidney injury	47 (27.6)	123 (72.4)	
	Gastrointestinal hemorrhage	7 (46.7)	8 (53.3)	
	Liver dysfunction	26 (8.4)	285 (91.6)	
	Acute respiratory distress syndrome	39 (37.5)	65 (62.5)	
**Hospitalization, staging, and outcome**				
COVID-19 stage				<0.001 ^a^
	1	18 (1.0)	1814 (99.0)	
	2	15 (1.4)	1033 (98.6)	
	3	5 (1.1)	461 (98.9)	
	4	21 (15.3)	116 (84.7)	
	5	22 (44.9)	27 (55.1)	
Duration of hospitalization (days)				0.003 ^a^
	<7	10 (1.1)	941 (98.9)	
	>7	71 (2.8)	2510 (97.2)	
Outcome				<0.001 ^b^
	Death	22 (40.7)	32 (59.3)	
	Discharged alive	59 (1.7)	3419 (98.3)	
Laboratory and radiology investigations				
C-reactive protein (CRP) (mg/L)				<0.001 ^a^
	<10	48 (1.5)	3212 (98.5)	
	10+	33 (12.1)	239 (87.9)	
Hemoglobin (g/dL)				<0.001 ^a^
	<12	32 (7.1)	417 (92.9)	
	>12	49 (1.6)	3034 (98.4)	
Platelet count (×10^9^/L)				0.006 ^b^
	<450	74 (2.2)	3362 (97.8)	
	>450	7 (7.3)	89 (92.7)	
Total white count (×10^9^/L)				<0.001 ^a^
	<11	61 (1.9)	3135 (98.1)	
	>11	20 (6.0)	316 (94.0)	
Neutrophil count (×10^9^/L)				<0.001 ^a^
	<8	59 (1.8)	3184 (98.2)	
	>8	22 (7.6)	267 (92.4)	
Lymphocyte count (×10^9^/L)				>0.999 ^b^
	<4	78 (2.3)	3286 (97.7)	
	>4	3 (1.8)	165 (98.2)	

^a^ Results derived using Chi-Square test; ^b^ Results derived using Fisher’s Exact test.

**Table 2:** Univariate and multivariate logistic regression analysis performed on demographic and clinical characteristics of COVID-19 patients with secondary and/or co-infections.

		Univariate		Multivariate	
		Odd Ratio (95% CI)	*p*-Value	Adjusted OR (95% CI)	*p*-Value
**Age, years**		1.06 (1.04–1.07)	<0.001 ^^‡^^	1.03 (1.01–1.05)	0.003 ^^‡^^
**Gender**	Female	reference			
	Male	1.02 (0.62–1.68)	0.95		
**Co-morbidity**	Asthma	2.06 (0.82–5.19)	0.13		
	Chronic kidney disease	16.40 (9.02–29.81)	<0.001 ^^‡^^	3.76 (1.92–7.38)	<0.001 ^^‡^^
	Hypertension	7.72 (4.93–12.11)	<0.001 ^^‡^^	2.10 (1.12–3.91)	0.02 ^^‡^^
	Neoplasm	2.86 (0.37–21.94)	0.31		
	Chronic hematology disease	7.18 (0.86–60.31)	0.07	14.55 (1.64–129.01)	0.02 ^^‡^^
	Obesity	5.49 (1.62–18.62)	0.006 ^^‡^^		
	Diabetes mellitus	8.93 (5.69–14.02)	<0.001 ^^‡^^	2.69 (1.52–4.79)	0.001 ^^‡^^
	Smoking	0.80 (0.37–1.76)	0.58		
	History of taking medications	4.55 (2.92–7.10)	<0.001 ^^‡^^		
**Days of hospitalization**	Less than 7 days of hospitalization	reference			
	More than 7 days of hospitalization	2.66 (1.37–5.18)	0.004 ^^‡^^		
**Symptoms**	Fever	4.87 (3.07–7.71)	<0.001 ^^‡^^	2.21 (1.29–3.80)	0.004 ^^‡^^
	Cough	3.39 (2.16–5.31)	<0.001 ^^‡^^		
	Runny nose	1.12 (0.56–2.25)	0.76		
	Shortness of breath	7.29 (4.36–12.18)	<0.001 ^^‡^^		
	Vomiting/nausea	3.99 (1.55–10.24)	0.004 ^^‡^^		
	Diarrhea	4.23 (2.37–7.56)	<0.001 ^^‡^^		
	Skin rash	8.62 (1.00–74.59)	0.05		
**Complications**	Acute respiratory distress syndrome	48.37 (29.33–79.77)	<0.001 ^^‡^^	7.72 (3.94–15.12)	<0.001 ^^‡^^
	Stroke/cerebrovascular accident	44.20 (8.78–222.47)	<0.001 ^^‡^^		
	Congestive heart failure	12.75 (4.10–39.63)	<0.001 ^^‡^^		
	Cardiac arrhythmia	29.97 (13.65–65.81)	<0.001 ^^‡^^		
	Cardiac arrest	18.04 (7.31–44.50)	<0.001 ^^‡^^		
	DIVC	32.37 (10.05–104.28)	<0.001 ^^‡^^		
	Acute renal failure	37.40 (23.22–60.24)	<0.001 ^^‡^^	9.64 (5.17–17.99)	<0.001 ^^‡^^
	Gastrointestinal hemorrhage	40.71 (14.39–115.20)	<0.001 ^^‡^^		
	Liver dysfunction	5.25 (3.24–8.50)	<0.001 ^^‡^^		
**Outcome**	Discharged alive	reference			
	Death	39.84 (21.85–72.65)	<0.001 ^^‡^^		
**Stage of COVID-19**	1 Asymptomatic	reference			
	2 Symptomatic but no pneumonia	1.46 (0.73–2.92)	0.28		
	3 Pneumonia without hypoxia	1.09 (0.40–2.96)	0.86		
	4 Pneumonia with oxygen therapy	18.24 (9.46–35.19)	<0.001 ^^‡^^		
	5 Critically ill	82.12 (39.59–170.34)	<0.001 ^^‡^^		
**Chest X-ray**	Normal	reference			
	Abnormal	2.12 (1.36–3.29)	0.001 ^^‡^^		
**Systolic blood BP**	<140	reference		reference	
**(mmHg)**	>140	2.28 (1.45–3.60)	<0.001 ^^‡^^	2.02 (1.18–3.44)	0.01 ^^‡^^
**Diastolic BP**	<90	reference			
**(mmHg)**	>90	0.98 (0.49–1.98)	0.96		
**Respiratory rate**	<20	reference			
**(per minute)**	>20	9.63 (5.97–15.51)	<0.001 ^^‡^^		

Method (multivariate) = Backward Stepwise (Likelihood Ratio). Goodness-of-fit = Hosmer and Lemeshow test 0.448, Classification Table 97.7%, Nagelkerke R Square 17.9%. ^^‡^^ *p*-value < 0.05. DIVC, disseminated intravascular coagulopathy. BP, blood pressure.

**Table 3:** Difference in laboratory investigations among COVID-19 patients with and without secondary and/or co-infections.

Lab Investigations	Unit	With Secondary and/or Co-Infections		Without Secondary and/or Co-Infections		Total	Z Score *	*p*-Value
		*n*	Mean Rank	*n*	Mean Rank			
WBC count	(10^9^/L)	80	1870.79	3310	1691.26	3390	−1.621	0.105
Hemoglobin	(g/dL)	80	977.44	3310	1712.85	3390	−6.642	<0.001 ^^‡^^
Platelets	(10^9^/L)	80	1433.19	3310	1701.84	3390	−2.426	0.015 ^^‡^^
Neutrophil count	(10^9^/L)	79	2030.37	3268	1665.39	3347	−3.317	<.001 ^^‡^^
Lymphocyte count	(10^9^/L)	79	926.57	3301	1708.78	3380	−7.043	<.001 ^^‡^^
PCT	(ng/mL)	18	35.72	34	21.62	52	−3.194	0.001 ^^‡^^
LDH	(units/L)	44	2113.75	2397	1204.61	2441	−8.479	<0.001 ^^‡^^
D-dimer	(mg/L)	14	103.29	126	66.86	140	−3.202	0.001 ^^‡^^
Ferritin	(µg/L)	20	152.45	180	94.73	200	−4.231	<0.001 ^^‡^^
CRP	(mg/dL)	81	2296.37	3416	1736.02	3497	−5.073	<0.001 ^^‡^^

^^‡^^*p*-value < 0.05. * Mann–Whitney test. WBC, white blood count. CRP, C-reactive protein. LDH, lactase dehydrogenase. PCT, procalcitonin.

## Data Availability

Restrictions apply to the availability of this data and data sharing is not applicable.
